# Asymptomatic Bacteriuria Caused by *Chromobacterium violaceum* in an Immunocompetent Adult

**DOI:** 10.1155/2015/652036

**Published:** 2015-10-04

**Authors:** Narayan Dutt Pant, Manisha Sharma, Saroj Khatiwada

**Affiliations:** ^1^Department of Microbiology, Grande International Hospital, P.O. Box 11796, Dhapasi, Kathmandu, Nepal; ^2^CIST College, Kathmandu, Nepal

## Abstract

Because of increasing antimicrobial resistance, the treatment of the asymptomatic bacteriuria is not considered except in specific circumstances like during pregnancy or before invasive urologic procedures. We are reporting a first case of asymptomatic bacteriuria caused by *Chromobacterium violaceum* in a 16-year-old male. With the reporting of the *C. violaceum* which is notorious for its high propensity for hematogenous dissemination causing fatal sepsis (with reported mortality rate up to 65–80%) if prompt proper treatment is not given, as causative agent of asymptomatic bacteriuria, it is recommended to treat the asymptomatic bacteriuria caused by this organism.

## 1. Introduction

Asymptomatic bacteriuria is the presence of a significant number of bacteria in the sample of properly collected urine from a person with no signs of urinary tract infection [1]. Its prevalence varies according to the age, sex, sexual activity, and presence of abnormalities related to genitourinary tract. The causative organisms are diverse and may include Enterobacteriaceae,* Pseudomonas aeruginosa*,* Enterococcus* species, and group B* Streptococcus* with* Escherichia coli* being the most common organism responsible [2]. Urinary tract infection caused by* Chromobacterium violaceum* has been rarely reported [3]. No cases of asymptomatic bacteriuria caused by this organism have been reported yet in the world literature and no cases of infection caused by* Chromobacterium violaceum* have been reported from Nepal. Here, we are reporting a case of asymptomatic bacteriuria caused by* Chromobacterium violaceum* in an immunocompetent adult of 16 years. To the best of our knowledge, it is the first case in which* C. violaceum* was identified as the cause of asymptomatic bacteriuria. Current guidelines suggest screening and treatment of asymptomatic bacteriuria only in specific circumstances like during pregnancy or before invasive urologic procedures [4]. Because of increasing antimicrobial resistance problem, the patients with asymptomatic bacteriuria should not be treated unless there is evidence of potential benefit [2]. With the reporting of the highly virulent organism like* Chromobacterium violaceum* which has high propensity of hematogenous dissemination causing fatal sepsis with reported mortality rate up to 65–80% [5], as the causative agent of asymptomatic bacteriuria, it is recommended to treat the asymptomatic bacteriuria caused by this organism.

## 2. Case Report

**Figure 1:**
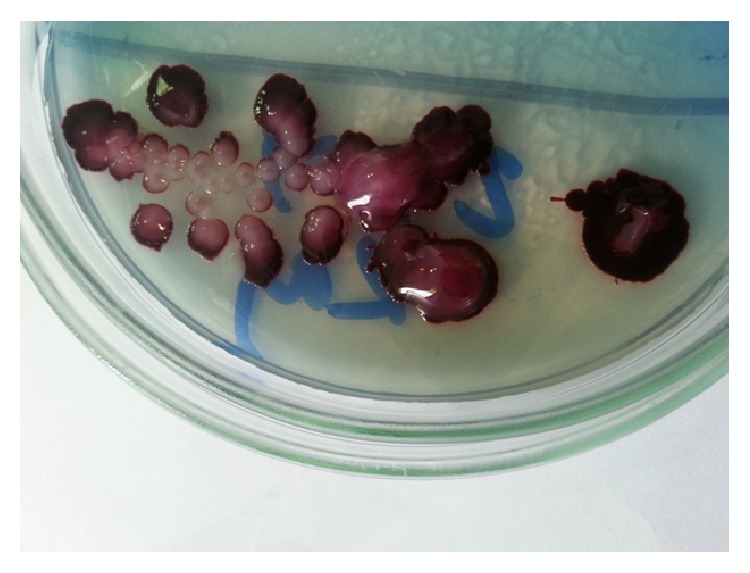
Growth of* Chromobacterium violaceum* in cystine lactose electrolyte deficient (CLED) agar.

A 16-year-old male attended the emergency department of a tertiary care hospital in Kathmandu, Nepal, in July 2015, with chief complaint of acute lower right abdominal pain. He has no past history of similar symptom but had a history of recurrent urinary tract infection in childhood and there were no urogenital tract abnormalities in the patient. He had a history of playing football and dancing in a field during a picnic, about two weeks before. By the time the patient reached the hospital, the symptom subsided spontaneously without any treatment. After physical examination the necessary laboratory investigations were requested. The abnormal laboratory findings reported were low polymorphs (38.2%), low eosinophils (0.8%), high lymphocytes (51.7%), and high mean corpuscular hemoglobin concentration (MCHC) (35.2%). Other biochemical and hematological investigations were normal (Tables 1 and 2). The tests like human immunodeficiency virus (HIV), hepatitis B surface antigen (HB_s_Ag), and diabetes screening were negative. No abnormality was detected in the ultrasonographical investigation of kidney and other abdominal organs but routine examination of urine revealed high leucocyte and erythrocyte counts with significant bacteriuria. Since the patient was asymptomatic, no treatment was given and we waited for urine culture and sensitivity report to come. The midstream urine culture plated on CLED agar showed significant (>10^5^ cfu/mL) growth of a single type of colonies after overnight aerobic incubation at 37°C. The colonies were 2 to 3 mm in diameter with violet nondiffusible pigment and they were round, convex, easily emulsible, glistening, and opaque (Figure 1). In Gram stain, the organism was Gram-negative bacillus. Conventional biochemical tests were performed by using standard microbiological tools and techniques as described in Bergey's manual of systematic bacteriology. The organism was motile rod, catalase, and oxidase producing and nitrate reducing. In triple sugar iron agar, it utilized glucose without producing gas and it did not grow in Simmons citrate agar. It did not produce urease, DNase, and indole and did not utilized sucrose, lactose, mannitol, and xylose but utilized fructose and trehalose. The bacterium hydrolysed gelatin and dihydrolysed the arginine but did not decarboxylyse lysine and ornithine and did not hydrolyse esculin. So on the basis of the above biochemical tests, Gram reaction, colony morphology, and pigment production, the organism was identified as* Chromobacterium violaceum*.

Antimicrobial susceptibility testing was performed by Kirby Bauer disk diffusion technique and the organism was found to be sensitive toward ceftriaxone, ciprofloxacin, cotrimoxazole, gentamicin, imipenem, norfloxacin, and piperacillin + tazobactam and resistant towards nitrofurantoin and amoxicillin + clavulanic acid. Since the organism is highly pathogenic with high propensity of dissemination causing fatal sepsis, blood culture was performed to rule out the hematogenous spread which was negative and the patient was treated with ciprofloxacin for one week as per urine culture and sensitivity report. After completion of the antibiotic course again, the urine culture was performed which was sterile.

## 3. Discussion

*Chromobacterium violaceum* is a Gram-negative bacillus that exists as a normal flora of water and soil mainly in tropical and subtropical regions. Despite its ubiquitous distribution, human infections are extremely rare, and there is limited awareness regarding the disease caused by this organism [6].* C. violaceum* was first identified in 1881; its pathogenic capability was first reported by Woolley in 1905, in a fatal infection of a buffalo [7], and the first case of human infection was noted in Malaysia in 1927 [8]. Since then, only 150 cases have been reported till 2007 in the world literature, with eight cases identified in the neighboring country India [9, 10].* Chromobacterium violaceum* is the only species of this genus responsible for causing human disease [11]. In human, it has been associated with respiratory tract infection, gastrointestinal infection, abscesses, meningitis, endocarditis, hemophagocytic syndrome, and fulminant sepsis [12]. The pattern of disease usually starts with a contaminated inoculation site, localized infection, regional lymphadenopathy, and then hematogenous dissemination to visceral organs [11]. Rapid progression to fatal sepsis with multiorgan failure is a characteristic feature of* Chromobacterium violaceum* infection [3].

But involvement of this organism in causing urinary tract infection has been rarely reported. Around only four cases of urinary tract infection caused by* Chromobacterium violaceum* have been found to be described in literature. No cases of asymptomatic bacteriuria caused by* C. violaceum* have been reported yet. It is considered as a bacterium of low virulence causing infection mainly in immunocompromised individuals [3] but in our case the patient was immunocompetent without any known predisposing factor. Urinary tract infection caused by* Chromobacterium violaceum* has also been described by Swain et al. [3] in an immunocompetent 19-year-old male but in contrast to our case the patient was symptomatic. Our patient was healthy without any history of conditions like chronic granulomatous disease (CGD), HIV, diabetes mellitus, or steroid therapy, which compromises the immunity and which must have contributed to preventing the development of the complications in case of our patient. The organism may get access into the body either through oral route by consumption of contaminated water or food or through exposure of damaged skin to stagnant water or soil. Unusual routes include infection after swimming in contaminated water [13], scuba diving or near drowning [14], and surgery [15, 16]. Infections have mainly been associated with contaminated recreational or stagnant muddy water [5]. But in our case, no similar history of the exposure could be determined. However, he had a history of playing football and dancing in a field during a picnic, about two weeks before, and he may have encountered the bacteria there.

*C. violaceum* is a mesophilic bacterium and infections by this organism are common in tropical and subtropical regions mainly in summer seasons [3, 11]. Infection caused by this organism has not been reported from Nepal. The main reason for this may be either due to poor health care system where the infection with the organism is underdiagnosed and hence unreported or due to temperature sensitivity of this organism; its geographic distribution changed with increasing global warming [9]. No study has been done to detect the presence of* Chromobacterium violaceum* in the environment of Nepal.

There are only a few conditions like pregnancy and before invasive urologic procedures in which antibiotic treatment of asymptomatic bacteriuria has been shown to improve patient outcomes and apart from these specific circumstances it is more beneficial not to treat asymptomatic bacteriuria due to increasing antibiotic resistance problem [2]. Our patient presented with pyuria accompanying asymptomatic bacteriuria which is not an indication for antimicrobial treatment [17].

But prognosis after establishment of the infection is very bad, with a mortality rate over 65% in localized infection and 80% in the cases of bacteremia [18]. It has high propensity to spread causing sepsis and death may take place in 1 week to 15 months from the time of infection [5]. So the prompt appropriate treatment was started as soon as the causative agent was isolated and its antimicrobial susceptibility pattern was known.

*C. violaceum* is usually sensitive toward chloramphenicol, tetracycline, gentamicin, cotrimoxazole, ciprofloxacin, and imipenem and resistant toward penicillins, cephalosporins, and aztreonam [19–21]. In a case report by Swain et al., the* C. violaceum* isolated from urine was found to be susceptible to ciprofloxacin, cotrimoxazole, imipenem, nitrofurantoin, and cefotaxime and resistant to amoxicillin + clavulanic acid [3]. In our study, the organism was sensitive toward ceftriaxone, ciprofloxacin, cotrimoxazole, gentamicin, imipenem, norfloxacin, and piperacillin + tazobactam and resistant toward nitrofurantoin and amoxicillin + clavulanic acid. Analysis of in vitro data suggests fluoroquinolones are the most active antibiotics against* C. violaceum* among all available antibiotics [22].

Since the organism was found to be sensitive towards ciprofloxacin, the patient was treated with this antibiotic for one week. Because of frequent relapse, long antimicrobial course and closed follow-up are necessary [11] but in our case the patient did not have any laboratory evidence along with symptoms of internal organ abscesses and dissemination of the infection the course for treatment given was only for one week and it was suggested that the patient come for follow-up as soon as possible if any suspicious symptoms appear.

Due to the high propensity of the* Chromobacterium violaceum* for hematogenous dissemination causing fatal sepsis, the importance of early diagnosis and proper antimicrobial therapy for the proper management of the ailment can never be underestimated. In our case, the patient did not develop any complication due to prompt diagnosis and timely administration of antimicrobial therapy as suggested by urine culture and sensitivity report.

## 4. Conclusion

Due to its ubiquitous presence in the environment, most of the time* Chromobacterium violaceum* may be disregarded as mere saprophytic contaminant even when it grows in clinical samples. This organism rarely causes infection and most of the clinicians are not aware of the disease caused by it. So the laboratory personnel along with the clinicians should be aware of the fact that human infection with* C. violaceum* is so rare but does occur and if timely proper treatment is not given it may rapidly progress to fatal septic shock.* C. violaceum* may also be responsible for causing asymptomatic bacteriuria and it is recommended to treat the asymptomatic bacteriuria caused by this organism to avoid fatal outcome.

## Figures and Tables

**Table 1:** Blood chemistry results of the patient.

Tests	Results	Reference range
Glucose random	104 mg/dL	70–140 mg/dL
Urea	21 mg/dL	15–45 mg/dL
Creatinine	0.8 mg/dL	0.5–1 mg/dL
Sodium	138 mmol/L	135–150 mmol/L
Potassium	4.4 mmol/L	3.5–5 mmol/L
C-reactive protein	3 mg/dL	0–10 mg/dL

**Table 2:** Hematology results of the patient.

Tests	Results	Reference range
Hemoglobin	15 gms%	13.5–16.9 gms%
Total leucocytes count	6250 cells/mm^3^	4000–11000 cells/mm^3^
Differential count		
Polymorphs	38.2%	40–70%
Lymphocytes	51.7%	20–45%
Eosinophils	0.8%	1–6%
Monocyte	9%	2–15%
Basophils	0.3%	<1%
Packed cell volume	42.6%	40–50%
RBC count	5.05 millions/mm^3^	4.44–5.61 millions/mm^3^
MCV	84.4 fL	81.8–95.5 fL
MCH	29.7 pg	27–32.3 pg
MCHC	35.2 g/dL	32.4–35 g/dL
Platelet count	3.06 lacs/mm^3^	1.5–4.5 lacs/mm^3^
